# Further considerations about “Detection and identification of enteroviruses circulating in children with acute gastroenteritis in Pará State, Northern Brazil (2010–2011)”

**DOI:** 10.1186/s12985-021-01600-5

**Published:** 2021-07-15

**Authors:** Raiana Scerni Machado, Ivanildo Pedro de Sousa, Jacqueline Cortinhas Monteiro, James Lima Ferreira, Jainara Cristina dos Santos Alves, Fernando Neto Tavares

**Affiliations:** 1 Laboratório de Referência Regional em Enteroviroses, Seção de Virologia, Instituto Evandro Chagas, Ananindeua, Pará Brazil; 2 Laboratório de Enterovírus, Instituto Oswaldo Cruz, Fundação Oswaldo Cruz, Rio de Janeiro, Brazil; 3 Laboratório de Virologia, Instituto de Ciência Biológicas, Universidade Federal Do Pará, Belém, Pará Brazil

**Keywords:** Enterovirus, Acute gastroenteritis, Brazil

## Abstract

On the detection and identification of enteroviruses circulating in children with acute gastroenteritis in Brazil: reply to Luchs, A. Comments on Detection and identification of enteroviruses circulating in children with acute gastroenteritis in Pará State, Northern Brazil (2010–2011).

**Dear Editor**,

We appreciate the opportunity to respond to the comments of Dr. Adriana Luchs, Adolf Lutz Institute, Brazil, on our recent paper published in the Virology Journal [1].

It is well-known that infections by enterovirus (EV) species are very common in the human population and are associated with a wide spectrum of clinically distinct syndromes affecting multiple body systems. Moreover, a high percentage of EV infections are asymptomatic. Although not recognised as medically important viruses causing acute gastroenteritis (AGE), the diarrhoeic potential of certain EV-types has been postulated in a number of surveillance studies [2–5]. Nonetheless, the role of EVs in the aetiology of AGE (including the newly recognised Picornaviruses Cosavirus and Parechovirus) still remains unclear since the vast majority of these earlier studies focussed solely on diarrhoea-causing enteropathogens without inclusion of matched healthy controls. Therefore, we fully agree with Dr. Luchs’ comments on our paper in that “Detection of EV strains exclusively must be interpreted cautiously”.

It is worth mentioning that rather than assessing a possible causative relationship between EVs and AGE cases among hospitalised children, our study sought mainly to improve our knowledge on the genetic diversity of EVs circulating in our region. In this regard, as a Regional Reference Centre for Enteroviruses of the Brazilian Ministry of Health, we believe that our investigation was conducted in compliance with the scope of the Global Enterovirus Surveillance Program, as part of the WHO and National Public Health responses in support to the Polio Eradication Initiatives.

It is mentioned in *Commentary VIRJ-D-21-00011* that “…none or the references cited [6] by Machado et al. stated the association of EV with acute diarrhea etc.” In this regard, it should be noted that such references (some of which suggested by our paper’ reviewers) related in fact to studies that reported the occurrence of EVs in the context of AGE—and other clinical syndromes—rather than establishing EVs as true diarrhoeagenic pathogens.

We thank Dr. Luchs for her considerations regarding a comparison of neutralising antibody titres in acute and convalescent phase (paired) serum specimens, so that serological diagnosis of EV could also be achieved. This would certainly be suitable in light of classical virological procedures. Currently, however, EV serological-based tests seem more relevant to epidemiological investigations than to clinical diagnosis, since the use of molecular biology methods in clinical virology has significantly changed approaches towards laboratory diagnostics of EV infections. Furthermore, we have essentially followed recommendations provided by WHO in the Enterovirus Surveillance Guidelines for Enterovirus Surveillance in Support of the Polio Eradication Initiative [7], where stools remain the clinical sample of choice for EV diagnosis.

As can be noted in our short report published recently in the Virology Journal, we have essentially searched for EVs in stool samples which had previously tested negative for other potential viral enteropathogens including rotavirus, parechovirus etc. Yet, even in cases where these samples were found to be EV-positive we precluded from highlighting any aetiological relationship between these pathogens and AGE. Notably, however, we were able to demonstrate a relatively high enterovirus detection rate among AGE patients which appears not being commonly observed. In addition, our study results were consistent with previous reports where a very high proportion of co-circulating non-poliovirus enteroviruses (NPEVs) were demonstrated, particularly those strains belonging to the human EV species C [6]. Of note, this might potentially give rise to recombination events which are known to often precede the emergence of novel evolutionary lineages of EVs. Further, we agree in that carefully designed studies are warranted to more accurately elucidate the role of EVs in the aetiology of AGE. These might typically be matched case–control studies where clinical and epidemiological data could be gathered, as well as data on the occurrence of other potential viral, bacterial and parasitic gastrointestinal pathogens.

## Conclusion

Overall, the establishment of a correlation between NPEV and AGE is complex. Further studies must be conducted to refine methods of detecting both ongoing and recent infections in patients with AGE to establish a causal link with NPEV. Finally, we believe that the information provided in our short report essentially highlights the need for continuous monitoring of EVs in cases of AGE, which represents a potentially valuable strategy towards strengthening surveillance for non-polio EV system in Brazil.

## Data Availability

Not applicable.

## Abbreviations

AGE: Acute gastroenteritis
EV: Enterovirus
WHO: World health organization
